# Resolution of lateral acoustic space assessed by electroencephalography and psychoacoustics

**DOI:** 10.3389/fpsyg.2013.00338

**Published:** 2013-06-11

**Authors:** Jan Bennemann, Claudia Freigang, Erich Schröger, Rudolf Rübsamen, Nicole Richter

**Affiliations:** Faculty of Biosciences, Pharmacy and Psychology, Universität LeipzigLeipzig, Germany

**Keywords:** auditory space processing, event-related potentials, mismatch negativity, minimal audible angle, spatial resolution, sound localization

## Abstract

The encoding of auditory spatial acuity (measured as the precision to distinguish between two spatially distinct stimuli) by neural circuits in both auditory cortices is a matter of ongoing research. Here, the event-related potential (ERP) mismatch negativity (MMN), a sensitive indicator of preattentive auditory change detection, was used to tap into the underlying mechanism of cortical representation of auditory spatial information. We characterized the MMN response affected by the degree of spatial deviance in lateral acoustic space using a passive *oddball* paradigm. Two stimulation conditions (SCs)—specifically focusing on the investigation of the mid- and far-lateral acoustic space—were considered: (1) 65° left standard position with deviant positions at 70, 75, and 80°; and (2) 95° left standard position with deviant positions at 90, 85, and 80°. Additionally, behavioral data on the minimum audible angle (MAA) were acquired for the respective standard positions (65, 95° left) to quantify spatial discrimination in separating distinct sound sources. The two measurements disclosed the linkage between the (preattentive) MMN response and the (attentive) behavioral threshold. At 65° spatial deviations as small as 5° reliably elicited MMNs. Thereby, the MMN amplitudes monotonously increased as a function of spatial deviation. At 95°, spatial deviations of 15° were necessary to elicit a valid MMN. The behavioral data, however, yielded no difference in mean MAA thresholds for position 65 and 95°. The different effects of laterality on MMN responses and MAA thresholds suggest a role of spatial selective attention mechanisms particularly relevant in active discrimination of neighboring sound sources, especially in the lateral acoustic space.

## Introduction

The accurate localization of single sound sources in everyday complex acoustic environments is a difficult task. In principle, sound localization relies on the processing of feature differences at both ears (binaural cues) and spectral filtering characteristics of the outer ear (monaural cues). Binaural cues are interaural time (ITD) and intensity differences (IID) caused by different sound arrival times and intensities at the two ears varying with spatial position of the sound sources (Middlebrooks and Green, 1991; Blauert, 1996). According to the “duplex theory” localization of low frequency sounds (<1.5 kHz) is mainly based on the processing of ITDs, whereas localization of high frequency sounds relies on the processing of IIDs. A large body of evidence suggests that the cortex is essential in auditory spatial cognition. In animal studies it has been shown that bilateral ablation of the auditory cortices leads to the inability to approach a sound source (Jenkins and Merzenich, 1984; Heffner, 1997). In humans, hemispheric brain damage in the respective areas generally causes deficiencies in spatial behavioral tasks (Zatorre and Penhune, 2001; Spierer et al., 2009) with more severe consequences following right hemispheric lesions. But it is important to consider that the encoding of spatial information at the cortical level depends on multifaceted monaural and binaural, hierarchically organized signal processing in the ascending auditory pathway. Generally, location cues (ITDs and IIDs) for sound source processing are extracted in nuclei of the superior olivary complex which send converging information to the inferior colliculus in the midbrain (reviewed in Grothe et al., 2010). To date, it is unclear in which way location information is encoded in the auditory cortex and how spatial acuity finds a neuronal representation or in which way the evoked activity for two distinct sound sources differs. Since there is hardly any evidence for topographical spatial representations within the auditory cortex, recent studies put forward the hypothesis of a populations rate code organized in opponent channels broadly tuned to the left and right hemifield (Phillips, 2008; Salminen et al., 2009, 2010; Magezi and Krumbholz, 2010; Briley et al., 2012). Further consideration suggest the involvement of four channels: two—a contralateral and ipsilateral—for each hemisphere (Stecker et al., 2005), whereas the balance in activation of these channels may differ between hemispheres (Krumbholz et al., 2005; also discussed in Magezi and Krumbholz, 2010).

In the present study we use electroencephalography (EEG) and psychoacoustics to investigate auditory acuity in space processing in the lateral acoustic field with respect to both, sound source detection thresholds and discrimination thresholds.

The evaluation of the event-related potential (ERP) mismatch negativity (MMN) helps to step into the nature of sound source representation by disclosing (preattentive) deviance detection mechanisms (Schröger, 1997; reviews: Kujala et al., 2007; Näätänen et al., 2007; May and Tiitinen, 2010). The major sources responsible for MMN were found in primary and secondary auditory cortical areas as well in frontal cortical areas (for review see Picton et al., 2000), while the exact neural generators seems to alter specifically with stimulus property, i.e., distinct MMN generators for deviance in intensity, frequency, and duration (e.g., Giard et al., 1995). A large body of evidence suggests that the MMN is a reliable tool to assess the resolution of acoustic feature processing including spatial acuity (Deouell et al., 2006; Pakarinen et al., 2007; Vaitulevich and Shestopalova, 2010).

For static acoustic stimuli differing in their location, the MMN (location-MMN) reaches peak values 100–250 ms after stimulus onset (Näätänen and Winkler, 1999; Deouell et al., 2000) and with signal onset as early as 94 ms for acoustic stimulations in frontal positions (Deouell et al., 2006). The latter study also reported an acuity of at least 10° in location-MMNs recorded for acoustic standards at 5° (within the right hemifield) and a linear relation between the amount of spatial deviation and MMN amplitude [*magnitude of deviation (MoD) effect on location MMN*]. Additionally, the use of source localization analysis enabled to assign the neural generator of location-MMN to the posterior superior temporal gyrus (planum temporale, PT, Deouell et al., 2006) which is known as site of auditory spatial processing in humans (e.g., Krumbholz et al., 2005; Altmann et al., 2007). Location-MMNs were also reliably recorded with standards at more lateral positions (Colin et al., 2002; Röttger et al., 2007), though only for spatially more separated deviants. Paavilainen et al. (1989) systematically quantified MMN amplitudes for different azimuthal sound source positions, and found no relation between stimulus deviance and MMN amplitude. These results were interpreted as an indication of an “all-or-none” phenomenon, i.e., different spatial deviations elicit MMNs with comparable amplitudes. However, this is not in agreement with headphone studies, in which different lateralities were simulated by systematically varying ITDs and where MMN amplitudes increased with larger ITD differences (Paavilainen et al., 1989; Nager et al., 2003; Pakarinen et al., 2007; Vaitulevich and Shestopalova, 2010).

Tests using behavioral localization tasks revealed the highest localization precision for center positions and gradual declines with increasing laterality (Stevens and Newman, 1936; Oldfield and Parker, 1984; Middlebrooks and Green, 1991; Recanzone et al., 1998; Savel, 2009). Thus, behavioral data in healthy subjects suggest a fine-grained resolution of acoustic space in human auditory cortex: For broadband noise stimuli the location error (“localization blur,” Middlebrooks and Green, 1991; Blauert, 1996) is about 5° in the frontal acoustic field and increases up to 20° for lateral positions. Another aspect in auditory space processing is spatial acuity, i.e., the ability of listeners to discriminate between two adjacent sound sources (minimal audible angle, MAA; Mills, 1958; Perrott, 1984; Hartmann and Rakerd, 1989; Perrott and Saberi, 1990). Mills (1958) reported values as small as 1° for pure tone stimuli presented in the front and 7° for peripheral positions at 75°.

From behavioral data we know that auditory localization accuracy and acuity in active localization and discrimination tasks declines for stimuli at lateral positions, but it is still unknown how accurate the lateral acoustic space is represented at the level of the auditory cortex. The present study aims at scrutinizing the resolution of lateral acoustic space as reflected by location-MMN, specifically focussing on possible *MoD*-effects and *laterality*-effects in MMN amplitude and latency. For this, we examined the automatic (preattentive) processing of spatial changes at behavioral subthreshold and near-threshold levels with deviations of 5, 10, and 15° within (1) the mid-lateral acoustic hemifield, i.e., relative to a 65° standard position and (2) in the far-lateral acoustic hemifield close to the interaural axis, i.e., 95°.

Additionally, the minimum audible angles (MAA, cf. Mills, 1958) were measured for the respective standard positions (65 and 95°) to reexamine the link between location-MMN and behavioral data, i.e., between cortical responses and behavioral localization blur across the azimuthal plane. If in the far-lateral acoustic space up to and beyond the position of the interaural axis the location of sound is automatically encoded with high spatial acuity then *MoD*-effects on location-MMN are to be expected, i.e., increased MMN amplitudes as a function of increased magnitude of sound sources deviation (as measured by Deouell et al., 2006 for the central acoustic space). If, however, at preattentive levels the cortical representation of mid- and far-lateral sound sources is blurred in comparison to sounds sources in the frontal plane (e.g., Paavilainen et al., 1989), then no *MoD*-effects on location-MMN are expected and/or only sound source deviations at behavioral threshold level, i.e., 10 and/or 15° are expected to elicit significant MMN responses. Furthermore, as inferred from respective behavioral data showing a gradual decrease in spatial resolution with increasing laterality (e.g., Mills, 1958), (1) generally higher MMN amplitudes and/or shortened MMN latencies are expected for deviations at mid-lateral positions (65°) than at far-lateral positions (95°) and (2) larger behavioral MAA thresholds for far-lateral (95°) than mid-lateral (65°) positions, respectively.

## Materials and methods

### Subjects

The MMN study was performed on 17 healthy subjects, aged 23–30 years (mean age ± *SD* = 24.2 ± 2.8 years; 12 females). The behavioral experiment on MAA was performed on additional 17 subjects (mean age ± *SD* = 26.4 ± 2.1 years; 8 females). All subjects gave written consent to take part in this study and were compensated for their expenses. The experiment was approved by local Ethics Committee of the Leipzig University and was conducted in accordance with the Declaration of Helsinki.

### Free field and setup

The acoustic free field laboratory is installed in a sound-attenuated, anechoic room (5.8 × 7.9 m; Industrial Acoustics Company, IAC, Niederkrüchten, Germany).

**Figure 1:**
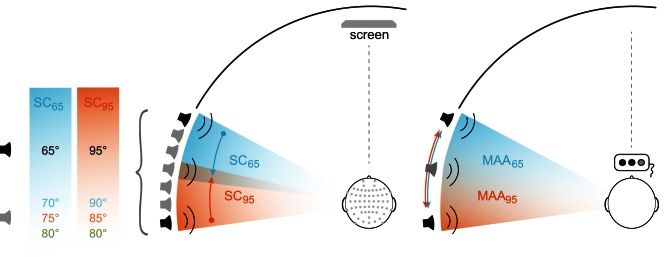
**Stimulus design**. Experimental setting for EEG (left) and MAA (right) experiments. Two different conditions (blue and orange) were tested differing in the position of the standard/reference stimulus (−65 or −95°, black loudspeaker symbols). In the EEG experiment a passive oddball-paradigm was used. Three location-deviant signals were presented in each condition (gray loudspeaker symbol) with the deviant position −80° being part of both experimental blocks. For MAA measurements, stimulus triplets composed of two reference stimuli and one test stimulus were presented with the position of the test stimulus randomly altered between the trials. Starting with a maximum deviation of 25°, the MAA was quantified using an adaptive 1up/1down procedure.

Seven broad-band loudspeakers (Visaton, FRS8 4Ω) were mounted at ear level in a semicircular array at 65, 70, 75, 80, 85, 90, and 95° within the left acoustic hemifield and with the 90°-position located on the subject's interaural axis (Figure 1). A comfortable, fixed chair was positioned in the middle of the semicircle at a constant distance of 2.35 m to the loudspeakers, such that subjects were aligned straight ahead to 0° azimuth (central position). The speakers indicated above were part of an array of speakers spanning the frontal semicircle from 98° to the left to 98° to the right. The complete array was covered by acoustically transparent, black gauze, so the subjects had no visual cue to determine the position of the loudspeakers. Each loudspeaker was equilibrated individually. For this, the transmission spectrum was measured using the a Bruel & Kjaer measuring amplifier (B&K 2610), a microphone (B&K 2669, pre-amplifier B&K 4190), a real-time signal processor [RP 2.1, System3, Tucker Davis Technologies (TDT)]. For each loudspeaker a calibration file was generated in Matlab 6.1. (The MathWorks Inc., Natick, MA, USA) and later used to ensure presentation of acoustic stimuli with flat spectra across the frequency range of the stimuli. The acoustic stimuli were generated digitally by a real-time processors of TDT (RX8 System 3, Tucker-Davis Technology, Alachua, FL, USA). Stimulus generation and experimental procedure were programmed in Matlab 7.5 (The MathWork Inc., Natick, MA, USA).

### Acoustic stimuli

The acoustic stimuli were low-frequency Gaussian white noise bursts (bandpass filtered at 300–1200 Hz) of 250 ms duration (including 10 ms cos^2^-ramps), separated by variable interstimulus intervals, 350–450 ms in the EEG recordings and 500 ms in the MAA measurements. It has been shown that the contribution of head movements to sound source localization of low frequency tones is restricted to stimulus durations >300 ms (Middlebrooks and Green, 1991; Blauert, 1996). Previous studies report that head motion elicited by an acoustic signal begins after 350 ms on average (reviewed in Blauert, 1996). It was also shown that for stimuli of 300 ms duration no differences in localization abilities were found between conditions where the head was mechanically immobilized or not (Thurlow and Mergener, 1970). In the present study such confounding effects of head movements were controlled for by employing 250 ms low-frequency noise bursts. Prior to the experiment, the subjects' individual hearing thresholds for the noise band were measured using a detection task (heard/not-heard paradigm). Based on this data, stimulus intensity was set at 40 dB SL (sensation level; values above individual hearing threshold) during the EEG recordings and the behavioral measurements. In the EEG recordings a level rowing of ±6 dB was used to prevent MMN components evoked by loudness cues due to minor differences in the position or orientation of the subjects' head.

### Experimental procedure for MMN acquisition

MMN was measured using a passive oddball paradigm. Acoustic stimulus presentation was organized in blocks of 2000 stimuli with standard stimuli occurring with a probability of 85% and the three deviant stimuli with a probability of 5% each. The experiment comprised two *stimulation conditions* (SCs) with standard locations at mid-lateral position (65°) and at far-lateral position near the interaural axis (95°) in the left acoustic hemifield (SC_65_ and SC_95_). SC_65_ and SC_95_ were tested in separate stimulation blocks including deviants with triple-tiered *MoD* [5°, 10°, 15°]: i.e., for SC_65_, infrequent deviant stimuli occurred at 70, 75, and 80°; respective deviant stimuli in SC_95_ were presented at 90° (i.e., on the interaural axis), 85, and 80°. For SC_65_, the deviants were shifted toward the periphery, for SC_95_ toward the center (Figure 1). Standards and deviants were presented in a pseudo-randomized manner with subsequent deviants interspersed by three standards at least.

During testing, subjects watched a subtitled movie on a LCD screen placed centrally at 0° azimuth. They were instructed to ignore the acoustic stimulation. During the experiments, subjects were supervised with an infrared camera from a control room. The entire experiment lasted about 45 min.

### EEG recordings

EEG was recorded with the Brain Product system (actiCAP, Brain Products GmbH, Munich, Germany). The EEG was recorded from Ag/AgCl active electrodes at 60 scalp positions according to the international 10–10 system (American Electroencephalographic Society, 1994). Recordings were referenced online to an electrode placed on the tip of the nose. The ground electrode was placed at position Fpz. Two additional electrodes were placed on the left and right mastoid. The vertical and horizontal electrooculograms (EOGs) were recorded to monitor eye movements. During the measurements, the impedance of scalp and eye electrodes was kept below 10 kΩ; the recordings were digitalized online at a sampling rate of 500 Hz.

### Analysis of EEG data

Offline data analysis was done using Matlab and EEGLAB (version 10.2.2.4b; Delorme and Makeig, 2004). The recordings were bandpass filtered at 1–20 Hz (1813 point Kaiser windowed sinc FIR filter, Kaiser beta = 5.65, firfilt plugin version 1.5.3. http://www.uni-leipzig.de/~biocog/content/widmann/eeglab-plugins/). For each trial, epochs of 600 ms duration including a 100 ms prestimulus baseline were analyzed. Epochs with amplitude changes exceeding 100 μV in any EEG or EOG channel were rejected from further analysis. Each epoch was referenced to the mean of the 100 ms prestimulus period (baseline correction). Individual ERPs were averaged separately for each SC and deviant type. Difference waves were calculated by subtracting ERPs to standards from ERPs elicited by respective deviant stimuli. Grand averages for each stimulus condition (SC_65_, SC_95_) and deviant type (5°, 10°, 15°) were computed separately from the averages of individual subjects. Before analyzing the MMN component, the difference waves were re-referenced to the mean ERP from the left and right mastoid, which increases the MMN signal-to-noise ratio because of the inversion of the MMN polarity at electrodes below the Sylvian fissure (Kujala et al., 2007).

To test for statistical significance of the MMN signal, one sampled Student's *t*-test was employed based on individual mean MMN amplitudes measured within a window of ±10 ms around the peak latency of the corresponding grand averaged response. The test was performed for each electrode site separately, in order to evaluate the potential distribution of MMN (cf. Figure 3). MMN signals that failed to reach the significance level were excluded from further analyses. For analyzing the effect of sound source *laterality* investigated in either of SCs and MoD on MMN amplitude and latency, the fronto-central electrode site (Fz) was pre-selected. MMN latencies were measured from the individual difference waves at the peak amplitude in a time window of 100–300 ms after stimulus onset. For each subject and deviant position, the MMN amplitudes were obtained as individual mean values within a time window of ±10 ms around the individual peak latencies. Statistical analysis of differences in MMN amplitudes and latencies across subjects were performed by repeated measurement (*rm*) analyses of variance (ANOVA) including factor *SC* [two levels: mid-lateral position at 65° (SC_65_), far-lateral position at 95° (SC_95_)] and factor MoD (three levels: 5, 10 and 15°). Significance was assessed by *post-hoc* paired comparison *t*-tests (with *Bonferroni* correction). Statistics on MMN data were done using the R environment (version: 2.10.1, The R Foundation for Statistical Computing).

A fine-grained resolution of lateral acoustic space for both (1) at mid-lateral position 65° and (2) at the far-lateral position 95° would be reflected by a significant main effect of factor *MoD* with increased MMN amplitudes and/or shortened MMN latencies as a function of increasing deviation (5° < 10° <15°).

A blurred cortical representation of far-lateral positions and positions around the interaural axis would be reflected by (1) significant interaction of factors *MoD* and *SC* with no *MoD*-effect on MMNs for SC_95_ and/or (2) *per se* no MMN-elicitation in SC_95_.

Further, a putative *laterality*-effect on MMN would be reflected by a significant main effect of factor *SC* with generally higher MMN amplitudes for changes in sound sources in SC_65_ than in SC_95_. To exclude putative N1 effects (Schröger and Wolff, 1996), a running *t*-test was used to test for differences between ERPs evoked by standard and respective deviant stimuli in the N1 time window ranging from 80–130 ms relative to stimulus onset. A significant difference of five subsequent data points (corresponding to 10 ms; *p* < 0.05) was set as criterion to disclose a possible N1 effect. The significance level for this test was adjusted following a Bonferroni–Holm method for multiple comparisons.

### Acquisition of minimum audible angle

In order to quantify spatial discrimination in separating distinct sound sources at attentive behavioral level, a MAA experiment was conducted. The MAA was measured separately in two experimental blocks for the respective reference positions at 65 and 95° also used in the EEG experiment.

### MAA testing procedure

The same acoustic stimuli were used as in the EEG-experiment, except the interstimulus interval was set to 500 ms. The MAA was examined by applying a three-alternative forced-choice paradigm (3AFC) and using the 1up/1down rule aiming at the 50% probability level for a correct response (Green and Swets, 1988). In the 3AFC testing, subjects were asked to differentiate between two reference signals, i.e., signals coming from the same angular position and one test signal differing in the angular position, with the order of reference and test signals randomly altered within the stimulus triplets. Responses were given by pressing appropriate buttons on a response box. Reference positions were at 65 and 95° (Figure 1). At the start of each trial, the deviant sound was presented with a spatial disparity of 25° toward more lateral positions for the 65° reference and toward more medial positions for the 95° reference. Spatial disparity between reference and deviant sound was decreased after each correct response and increased in case of a false response (1up/1down procedure; step size = 2.1°). Any change from a correct to a false response or vice versa was marked as a turn point. A single test run was terminated after five turn points and the thresholds were calculated as the mean of the last four turn points. Subjects were instructed to face straight ahead and to stay in that position during all stimulus presentations. The subjects' position was permanently monitored by the experimenter via video stream from the test chamber.

### Analysis of MAA data

Mean MAA thresholds were assessed by averaging four turn points in the staircase procedure for each subject separately. In order assure that subjects met the requirements of the task subjects performed each block twice in a pseudo-randomized order. For further analysis averaged threshold of both testings for each stimulus condition were used. No subject showed a variation larger than the populations' standard deviation for each stimulus condition. Thresholds were analysized using two sample Students paired *t*-test (two-tailed). In order to compare the variance of both SCs a *F*-test was used with a confidence level of 0.95 for the confidence interval. All analysis was done using the R environment (version: 2.10.1, The R Foundation for Statistical Computing).

A blurred accuracy of localization for sounds with increasing laterality would be reflected by significantly enhanced MAA thresholds in SC_95_ than SC_65_ (according to a putative *laterality*-effect on MMN).

## Results

### Electrophysiological data

**Figure 2:**
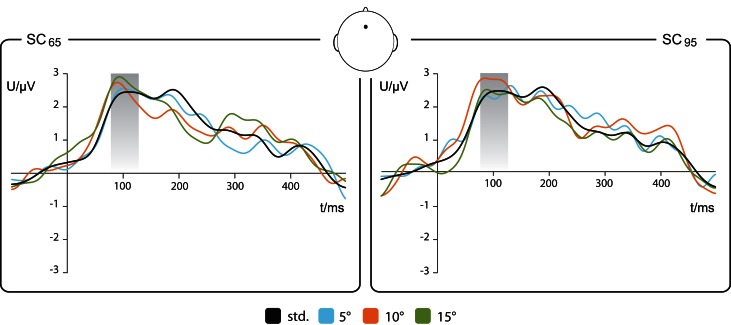
**Event-related potentials to standard and deviant stimuli**. Grand averaged EEG epochs recorded at Fz, see sketch of the view from above to the head. **Left**: stimulation condition SC_65_, 65° as standard (std), 70, 75, and 80° as deviants (−5, −10, and −15° deviation relative to std). **Right**: stimulation condition SC_95_, 95° as std, 90, 85, and 80° as deviants (+5, +10, and +15° deviation relative to std). Shadowed box depicts the (80–130 ms) N1 time window. The running *t*-test yielded no significant differences between standard and deviants (*n* = 17).

For both stimulus condition SC_65_ and SC_95_, ERPs were recorded for standard and deviant stimuli (Figure 2).

The ERPs evoked by standard and deviant stimuli showed a positive deflection in the EEG signal at about 100 ms after stimulus onset (comparable to Figure 2 of Deouell et al., 2006). The running *t*-test between ERPs evoked by standards and respective deviants revealed no significant differences within a time window of the N1 component ranging from 80–130 ms.

**Figure 3:**
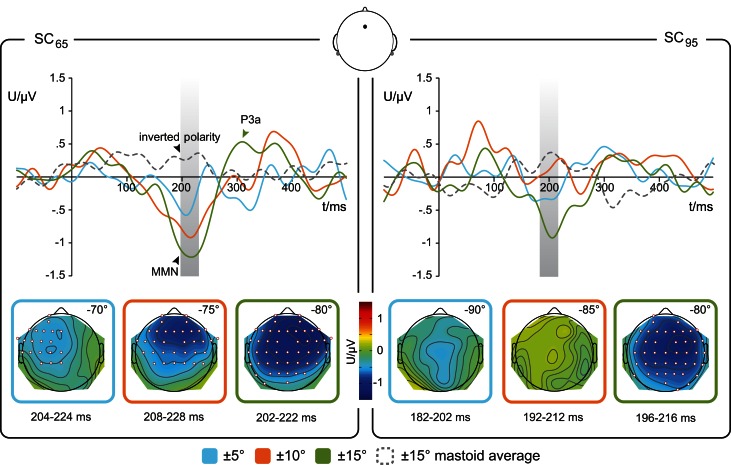
**Re-referenced difference waveforms**. Averaged difference waveforms (solid lines) at Fz re-referenced to mean ERP signal obtained at mastoid electrodes (dotted gray line). **Left**: deviations within the mid-lateral space (SC_65_) **Right**: deviations within the far-lateral space (SC_95_). The Magnitude of Deviation (MoD) relative to standard position is color-coded: blue ±5°, orange ±10°, green ±15°. Gray boxes indicate the 20 ms interval around the latency of the MMN peak in the re-referenced grand-averaged response which was used to test for statistical presence of MMN responses. Lower row: scalp distribution of the MMN amplitude for respective MoD, electrode sites with significant amplitudes (one-tailed *t*-test, *p* < 0.05) shown as red-edged dots; *n* = 17.

Difference waves were calculated from deviant-minus-standard ERPs. The MMN component was identified as a negative deflection with a reversed polarity at mastoid sites within a time window of 200 ± 50 ms after stimulus onset as exemplary demonstrated in Figure 3 (dotted line) for the Fz electrode and 15°-deviation in SC_65_ and SC_95_. MMN amplitudes and MMN latencies are listed in Table 1.

**Table 1:** **Mismatch negativity characteristics**.

**SC**	**MoD (deviant position)**	**Mean MMN amplitude (CI) [μV]**		**MMN latency (CI) [ms]**	
		**Within the time window of 20 ms around the individual peak MMN^1^**	**Within the time window of 20 ms around the GA peak MMN^2^**	**Mean of individual latencies^1^ (measured from the individual difference waves at the peak amplitude)**	**Analysis window^2^ (20 ms interval around the latency of the MMN peak in the re-referenced GA)**
SC_65_	5° (70°)	−1.07 (±0.58)	−0.54 (±0.58)^*^	211 (±12)	204–224
	10° (75°)	−1.75 (±0.82)	−0.89 (±0.72)^**^	199 (±15)	208–228
	15° (80°)	−2.02 (±0.63)	−1.20 (±0.74)^***^	209 (±13)	202–222
SC_95_	5° (90°)	−1.11 (±0.70)	−0.35 (±0.82)	199 (±15)	182–202
	10° (85°)	−0.71 (±0.69)	−0.09 (±0.73)	191 (±17)	192–212
	15° (80°)	−1.50 (±0.75)	−0.88 (±0.79)^**^	210 (±14)	196–216

1 Used for comparisons in repeated-measurement ANVOA, only for statistically significant MMN amplitudes.

2 Used to test for statistical significance of MMN signal (Student's t-test).

MMN amplitudes and latencies recorded at the fronto-central electrode site (Fz) for tested Magnitudes of Deviation (MoD) at respective deviant positions in both stimulation conditions, SC_65_ and SC_95_. MMN amplitudes and latencies are listed with upper and lower limit of the 0.95 confidence interval (CI). MMN amplitudes and latencies are depicted for both, the individual difference waves, and grand averaged difference waves (GA).

* p < 0.05,

** p < 0.01,

*** p < 0.001.

### Laterality effect on MMN

For the stimulus condition SC_65_ the one-sample *t*-tests (one-tailed) revealed significant MMNs for deviation magnitudes of 10 and 15°, most prominent at fronto-central electrode sites (Figure 3). For the 5°-deviants in SC_65_ the significant MMN component showed a leftward potential distribution covering central and left hemispheric electrodes (Figure 3, lower left). In SC_95_, only 15°-deviants elicited significant MMNs amplitudes. To test for a putative (*laterality*-) effect of the factor *SC*, MMN amplitudes, and MMN latencies elicited by a MoD of 15° were compared between SC_65_ and SC_95_. The two-sample *t*-test indicated a trend of higher MMN amplitudes for SC_65_ than for SC_95_ [*t*_(16)_ = −1.4393, *p* = 0.08]. The factor *SC* did not affect MMN latencies [*t*_(16)_ = −0.1642, *p* > 0.05].

### Magnitude of deviation-effect on MMN in SC_65_

**Figure 4:**
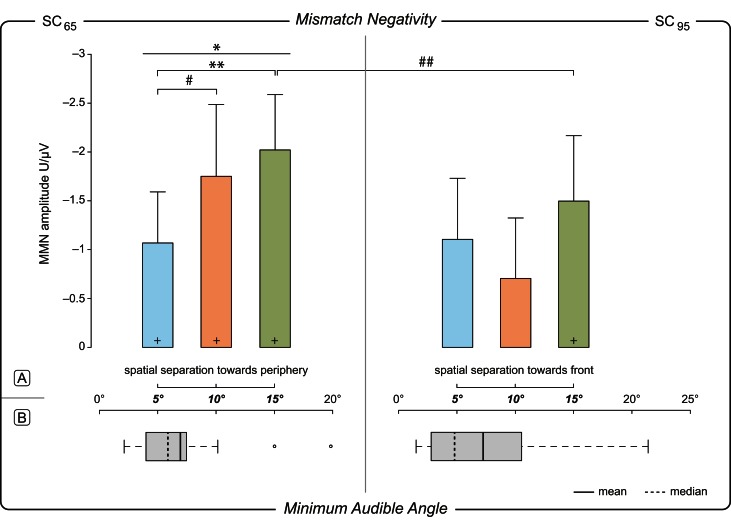
**Dependence of MMN amplitude on magnitude of deviation. (A)** *Mismatch Negativity*. Mean individual MMN amplitudes (*n* =17) within a ±10 ms time window around the individual MMN peak amplitude measured at electrode site Fz plotted against the Magnitude of Deviation, MoD, i.e., ±5°, ±10°, ±15° relative to 65° (left, SC_65_) and 95° (right, SC_95_) std position. Upper limit of 0.95 confidence intervals for the mean values are indicated as error bars. One-Way *rm*ANVOA revealed a significant *MoD*-effect on MMN amplitude [*F*_(2, 32)_ = 5.058, *p* = 0.012] in SC_65_. *Post-hoc* pairwise comparisons revealed higher amplitude for 15° deviation compared to 5° deviation and a tendency toward higher amplitudes for 10° compared to 5° deviation. ^*^/^**^ significance at a level of *p* < 0.05/*p* < 0.01; ^#/##^ tendency *p* = 0.09/*p* = 0.08; ^+^MMN amplitudes significantly different from 0. **(B)** *Minumum Audible Angle*. MAA plotted for both stimulation conditions 65° (left) and 95° (right). Mean values—solid line, median—dashed line. Whiskers extent to the most extreme data point which is within the range of 1.5 times of the interquartile distance (1st–3rd quartile). Outliers are shown by open dots. Interquartile distance of MAA thresholds (1st–3rd quartile): 3.5° for reference at 65°, and 7.8° for reference at 95°; *n* = 17.

For SC_65_ the *rm*ANOVA for individual MMN amplitudes (Table 1) showed a main effect of MoD [*F*_(2, 32)_ = 5.058, *p* = 0.012]. *Post-hoc* pairwise comparisons showed significant differences between the MMN amplitudes elicited by MoD 5° and MoD 15° [*t*_(16)_ = 3.395, *p* = 0.005] with larger MMN amplitudes for MoD 15° [MoD 5°: mean(±*SD*) = −2.02(±1.19) μV, MoD 15°: mean(±*SD*) = −1.07(±1.09) μV; Figure 4A]. The comparison of MMN amplitudes elicited by MoD 5° and by MoD 10° [mean(±*SD*) = −1.75(±1.54) μV] resulted in a trend [*t*_(16)_ = 1.98, *p* = 0.09] of higher MMN amplitudes for MoD 10° than for MoD 5°.

Further, in SC_65_ the factor *MoD* had no effect on MMN latencies [*F*_(2, 32)_ = 1.5, *p* > 0.05].

### Behavioral data

MAA thresholds measured for both lateralities, (1) mid-lateral position 65° and (2) far-lateral position 95° and shown in Figure 4B. For sound sources at 65° individual thresholds of MAA varied between 2.1 and 19.8° (*i.e*., −67.1° to −84.8°), and for 95° between 1.5 and 21.4° (*i.e*., −93.5° to −73.6°). However, the comparison of MAA thresholds obtained for both reference positions (MAA at 65° position: mean(±*SD*) = 6.96°(±4.6°); MAA at 95° positions: mean(±*SD*) = 7.27°(±6.1°) revealed no statistically significant difference [*t*_(16)_ = −0.28, *p* = 0.61, Figure 4B]. Although the interquartile distance for 95° seems to tend to be greater than for 65° the statistical analysis revealed no performance differences on a population level [*F*_(16)_ = 0.564, *p* = 0.26; Figure 4B].

## Discussion

Healthy human listeners are able to separate closely neighbored auditory events around the central midline with high precision (up to 1° Mills, 1958), while acuity performance gradually decreases with the laterality of sound sources (*e.g*., Blauert, 1996). The assignment of a spatial position to an acoustic object is regarded as a cortical achievement. This notion is substantiated by data from patients, who can show severe impairments in sound localization when suffering from auditory cortex lesions (e.g., Zatorre and Penhune, 2001). Based on results from animal studies, several models of neuronal population coding of auditory space were put forward, which reconcile the apparent low resolution at neuronal levels with the relative high precision in behavioral sound source localization (e.g., Middlebrooks et al., 1998; Stecker et al., 2005; Phillips, 2008; Magezi and Krumbholz, 2010). In numerous studies on healthy human subjects, MMN was used as an electrophysiological tool to explore the nature of neural resolution of cortical acoustic space representation and to quantify sound source detection abilities (Paavilainen et al., 1989; Schröger and Wolff, 1996; Colin et al., 2002; Nager et al., 2003; Altman et al., 2005; Deouell et al., 2006, 2007; Pakarinen et al., 2007; Röttger et al., 2007; Spierer et al., 2007; Richter et al., 2009; Vaitulevich and Shestopalova, 2010; Koistinen et al., 2012). In most of these studies, the acoustic space explored was restricted to the frontal areas, which sets limits when drawing general conclusions about cortical acoustic space representation. Here, we characterized location-MMNs affected by sound source deviations in lateral azimuthal space to provide better understanding of altered localization acuity at lateral position and their neuronal representation.

### Effect of magnitude of deviation on location-MMN

At 65°, a spatial deviation of 5° already elicited a noticeable MMN response, and with larger spatial deviations up to 15°, the location-MMN monotonously increased. These results imply a neural resolution of at least 5° for sound sources at mid-lateral positions (65°). Earlier studies on acoustic space representation based on the analysis of neuronal responses to acoustic free field sounds showed that the magnitude of spatial deviance is linearly tracked by MMN for the central acoustic field up to a laterality of 35° (Deouell et al., 2006). From these data, a spatial acuity of at least 10° was inferred, which is in agreement with human localization abilities measured in behavioral experiments (1–10°; Blauert, 1996). Other acoustic free field studies investigating MMN for more lateral stimulus positions failed to show a covariance of location-MMN amplitudes with the degree of deviation (Paavilainen et al., 1989; Colin et al., 2002). However, most studies dealing with azimuthal localization acuity are based on headphone stimulation investigating perceived sound source lateralization by varying ITDs (e.g., Nager et al., 2003; Vaitulevich and Shestopalova, 2010). Under such stimulus conditions the smallest ITDs that caused discerneable MMNs were 50 μs (Pakarinen et al., 2007) or even 20 μs (Vaitulevich and Shestopalova, 2010) corresponding to spatial changes of 5 or 2.25°, respectively. Still, the processing of a single acoustic cue, such as ITDs, cannot directly be equated with the processing of the full range of auditory spatial information available under free field conditions (Phillips, 2008). Thus, a valid interpretation of EEG data with respect to the cortical processing of spatial acoustic information has to clearly distinguish between internalized (intracranial) percepts of signal lateralization (i.e., varying ITDs under headphone stimulation, see also Schröger, 1996) and externalized perception of acoustic objects (i.e., signal presentation under free field conditions at different locations).

The present findings of high spatial acuity in the mid-lateral acoustic space reveal that MNN is a very sensitive tool for the investigation of spatial acoustic processing, and the current data extend the results of Deouell et al. (2006). Notably, the high sensitivity of MMN in the present study and also in the study by Deouell et al. (2006) might be due to the specific multi-deviant MMN paradigm used in these studies, i.e., three types of deviants were paired with one standard. More important, the results possibly indicate that respective preattentive processing not only encodes the deviation *per se*, but also conveys an evaluation of the magnitude of their spatial deviation in relation to the standard position (i.e., *small*, *medium*, and *large*). Such a spatial processing mechanism at a preattentive level might be important to actually assign auditory objects to their spatial locations (i.e., *object formation*). This in turn is a prerequisite for higher-order cortical processes, such as those involved in *cocktail party situations* (Alain and Arnott, 2000). The results obtained with the presently used MMN paradigm is not necessarily inconsistent with the findings by Paavilainen et al. (1989), who did not find any modulations of MMN amplitudes with increasing spatial deviations. In their experiments the standard stimuli (0°) were always paired with only one deviant presented in three successive blocks (30, 60, and 90°).

When interpreting the observed modulation of the MMN with spatial deviation, also a partial overlap of MMN and N1 has to be considered (shown for increasing pitch differences between standard and deviant: Horváth et al., 2008). The overlap of N1 and MMN potentials is referred to as the refractoriness-hypothesis, which claims that neural populations responsive to repetitive standard stimuli are more refractory than newly recruited neural populations responsive to deviant stimulus features (May and Tiitinen, 2010). In the present study, the MMN amplitudes peaked within 200–210 ms after stimulus onset and were maximal over fronto-central scalp areas with inverting polarity at mastoid electrode sites (Figure 3, dotted line). Typically, the N1 occurs earlier in the ERP, i.e., around 100 ms after stimulus onset (review: Näätänen and Picton, 1987). Thus, if at all, only a small contribution of the N1 component to the observed MMN amplitudes is to be expected (for review see Näätänen et al., 2005). Additionally, the statistical testing failed to show significant differences between standard and deviant ERPs in the N1-time window, which contradicts the interpretation of present MMN results simply in terms of the refractoriness-hypothesis.

But notably, while it was repeatedly shown that the degree of deviance and corresponding MMN amplitude correlate positively (see Näätänen et al., 2007, for review), Horvath and colleagues were able to show that the average MMN amplitude (in their case related to increasing pitch deviations) rather indexes the percentage of detected deviants, than the difference between neural stimulus representations of deviant and standard stimuli (Horváth et al., 2008). If this is considered, above threshold deviations have a higher probability of being detected than near threshold deviations. As a consequence, in the first case all instances will elicit MMNs, while in the second case the rate of MMNs will be lower, resulting in lower average MMN amplitudes (for elaboration of this idea cf. Winkler et al., 2001; Schröger et al., 2007; Horváth et al., 2008). In the present study the 15° deviation more consistently evoked MMN compared to the 10°, and the 5° deviation. This resulted in the observed *MoD-effect* on MMN amplitude. This hypothesis would also explain the findings by Paavilainen et al. (1989), who evaluated location-MMN related to spatial deviations well above behavioral thresholds.

It should not be concealed that the present MMN latencies were relatively long compared to those measured for sound sources within the central acoustic space (Deouell et al., 2006). Considering that sound source localization deteriorates toward the sides (Blauert, 1996), signals perceived from the far lateral space may require additional neural processing efforts, which in turn is reflected in a delay of cortical MMN activity (Röttger et al., 2007; Richter et al., 2009).

### Effect of sound sources laterality on location-MMN

For SC_95_—and different from SC_65_—spatial separations of 5° and 10° were not preattentively detected by mechanisms underlying the MMN. The evaluation of these differences must consider two particularities which come along with auditory space processing of signals in the horizontal plane. Firstly, ITDs non-linearly increase with lateral sound source eccentricity resulting in different ITD changes for identical angular shifts (Blauert, 1996). Secondly, around 90° laterally, binaural variations of angular shifts do not produce ITDs and IIDs termed “cone of confusion” (Shinn-Cunningham et al., 2000). Present signals from 85 and 90° do not substantially differ in their ITD from the 95° standard causing the failure to evoke discernable MMNs. Additionally, differences in spectral information due to changes in sound source laterality were only barely available because of the presently used low-frequency band pass noise bursts (cf. Röttger et al., 2007 using broad band stimuli including higher frequencies). Additionally, the stimulation of SC_65_ and SC_95_ condition differ in their direction of presented oddball shifts: in SC_65_ all deviant locations were located toward the acoustic periphery, while in SC_95_ the deviants were shifted toward the acoustic center (midline). A previous study could show that a so-called “change response” (cN1, cP2; transient response to the ITD change at the transition from the “adaptor” to the “probe” signal) is affected by the direction of that ITD change (Magezi and Krumbholz, 2010). The authors provided evidence that the cN1 and cP2 deflections are larger for outward changes compared to inward changes, which is in line with the “opponent channel” coding hypothesis (Magezi and Krumbholz, 2010 see also von Békésy, 1930; van Bergeijk, 1962; McAlpine et al., 2001; Salminen et al., 2009, 2010; Briley et al., 2012). Still, it is unclear to what extent the mechanism underlying the “change response” can be linked to the corresponding mechanism underlying the MMN response (Jones et al., 2000). Further, to our best knowledge, a respective correlation has not yet been evaluated for the MMN component. In a previous MMN-study, Richter et al. (2009) used a passive oddball paradigm with location deviants shifted 17° “toward front” and “towards periphery” relative to ±73°, and did not find directionality effects of the MMN. Interestingly, the results point to potential directionality effect on MMN in terms of differences in the hemispheric activity patterns, i.e., location deviants shifted toward the periphery evoked a contralateral predominance, while location deviants shifted toward the front led to similar activation patterns in both hemispheres. Also, Deouell et al. (2006) presented 10° location deviants to both sides of a reference sound at +5°, i.e., deviant locations were at −5° (toward acoustic center and crossing the midline) and +15° (shift toward acoustic periphery). The authors found no statistical difference in the MMN evoked by −15° and +15° location deviants. Despite the fact that the results of both MMN-studies do not favor a directionality effect on MMN amplitudes *per se*, further studies are needed to examine in more detail a putative relation between the direction of spatial displacement and MMN modulation/elicitation.

For mid-lateral 65°, the acuity of change detection in the passive listening experiment compare well with obtained thresholds in the behavioral MAA experiment. This confirms previous findings showing a close relationship between MMN sensitivity and behavioral thresholds, as reported for a large variety of acoustic stimulus features (Näätänen et al., 1993; Tiitinen et al., 1994; Näätänen and Alho, 1997; Amenedo and Escera, 2000). Strikingly, for the far-lateral reference position 95° the average MAA is around 7°. So, while subjects were able to consciously differentiate between sounds at 95 and 88°, this performance is not reflected in the MMN experiment, where deviations of 5 and 10° failed to elicit a MMN. As mentioned above, in many cases MMN sensitivity match behavioral threshold, but still it is not understood, in which way electrophysiological indicators of successful change detection correspond to detection and discrimination thresholds in behavioral experiments. Previous studies on the one hand reported MMNs at behavioral subthreshold levels (Allen et al., 2000; Paavilainen et al., 2007), while other studies documented the ability to discriminate changes in acoustic features without a preceding MMN (e.g., Pettigrew et al., 2004). In this connection, investigations of the relation between performance in target detection tasks and parameters (e.g., amplitude) of MMN yielded partially incongruent results (cf. Näätänen et al., 2007 and Schröger, 1997, for reviews): some studies showed in the course of discrimination training discernable MMN even before behavioral discrimination ability (Tremblay et al., 1998; Menning et al., 2000). Other studies found MMNs to acoustical changes which were not behaviorally discriminated (e.g., Alho and Sinervo, 1997; Allen et al., 2000; Paavilainen et al., 2007). In general, it seems that behavioral discrimination ability is partly governed by the preattentive discrimination process as reflected in MMN (Lang et al., 1990; Tiitinen et al., 1994; Amenedo and Escera, 2000). In the present study, the lack of linkage between MMN amplitude and the subjects' performance at SC_95_ may partly relate to the high level of difficulty (see section above, Horváth et al., 2008) in the localization tasks, i.e., localization performance around the “cone of confusion” (MAA, Blauert, 1996). But on the other hand one might also argue that threshold estimations using different psychophysical methods (e.g., yes-no methods or forced-choice methods) may require different levels of attention and thus result in significantly different results (Jäkel and Wichmann, 2006). The proposed convertibility of forced-choice thresholds to those measured by single interval methods claimed by signal detection theory formalism (Green and Swets, 1988) is still under debate (see also discussion section above).

However, the involvement of additional resources of selective auditory attention in the behavioral task may be considered as being important to preserve behavioral thresholds even at far-lateral positions close to the interaural axis. Based on previous findings it is known that focusing auditory spatial attention on a specific target direction significantly improves response performance (Mondor and Zatorre, 1995; Teder-Sälejärvi and Hillyard, 1998; Teder-Sälejärvi et al., 1999), with the density of resources of auditory attention gradually declining with distance from the specific attentional focus (“gradient model” of attention; Mondor and Zatorre, 1995; Arnott and Alain, 2002). Additionally, in a recent study Lee and Middlebrooks (2011) could show that the coding of spatial location in a hemifield code (according to the “opponent channel” model) can be adjusted depending on the task. Neurons of cat's primary auditory cortex showed different activation patterns during idle listening and while performing an auditory localization task. During listening, neurons were activated by a wide range of spatial location but became more spatially selective when the cat was engaged in the localization task, i.e., receptive fields of activated neurons became narrower. Instead of moving the head to increase spatial discriminability, the modulation of auditory neuronal activity in accordance to the requirements of the task might be a potential strategy to improve response behavior at a location of interest (Salminen et al., 2012, compare also Ahveninen et al., 2006). Taken together, this might explain the improved localization acuity in the behavioral experiment, in which subjects attentively listened to discriminate different sound locations, whereas in the MMN experiment subjects' attention was targeted toward the screen in front of them at central midline. Taken together, this **might** explain the improved localization acuity in the behavioral experiment for SC_95_, in which subjects attentively listened to discriminate different sound locations: under passive listening condition the position-depended small acoustic differences between deviant and standard position were not sufficiently large enough to trigger the MMN-generating system. But during active listening, as in present MAA experiment, additional attentional top-down resources could have been made available for the auditory system to discriminate such small acoustic difference more efficiently—necessary for accurate localization within the “cone of confusion.” Previous studies suggest that such recruitments of attention might improve the neuronal signal-to-noise ratio of encoded acoustic signals and enhance the concious information processing (Fritz et al., 2007; Okamoto et al., 2007).

Further research is needed to clarify the putative involvement of attentional resources in dissolving confused situations occurring in far-lateral acoustic space. In such studies, the role of attention during active sound source discrimination can be tested in an experiment similar to the present one, but with a within-subject design allowing for a direct correlation of MMN amplitudes with respective behavioral performance. Alternatively, the contribution of selective auditory attention on location-MMN could be further tested by using an oddball stimulation paradigm similar to the on in the present study but using attentive SC to specifically evaluate the correlation between MMN and behavior under preattentive vs. active sound source detection.

## Conclusion

The electrophysiological data showing auditory driven preattentive deviant detection and elicitation of MMNs triggered by location changes of sound sources provide evidence for considerably high location acuity within mid-lateral acoustic space: at a laterality of 65°, minimal spatial deviation of 5° elicited salient MMN responses. The MMN monotonously increased for spatial deviations of 10 and 15°. These results imply a neural resolution of at least 5° for sound sources in the mid-lateral space. Behavioral data suggest that the higher spatial acuity during active SCs at 95° may be due to deploying additional top-down attentional resources.

### Conflict of interest statement

The authors declare that the research was conducted in the absence of any commercial or financial relationships that could be construed as a potential conflict of interest.
